# ChiTaRS 8.0: the comprehensive database of chimeric transcripts and RNA-seq data with applications in liquid biopsy

**DOI:** 10.1093/nar/gkae1126

**Published:** 2024-12-16

**Authors:** Dylan DSouza, Lihi Bik, Olawumi Giwa, Shahaf Cohen, Hilit Levy Barazany, Tali Siegal, Milana Frenkel-Morgenstern

**Affiliations:** Azrieli Faculty of Medicine, Bar Ilan University, Henrieta Szold 8, Safed, 1311502, Israel; Scojen Institute of Synthetic Biology, Reichman University, Hauniversita 8, Herzliya, 4010101, Israel; Azrieli Faculty of Medicine, Bar Ilan University, Henrieta Szold 8, Safed, 1311502, Israel; Scojen Institute of Synthetic Biology, Reichman University, Hauniversita 8, Herzliya, 4010101, Israel; Scojen Institute of Synthetic Biology, Reichman University, Hauniversita 8, Herzliya, 4010101, Israel; Rabin Medical Center (Beilinson Campus), Zeev Jabotinsky St 39, Petah Tikva, 49100, Israel; Azrieli Faculty of Medicine, Bar Ilan University, Henrieta Szold 8, Safed, 1311502, Israel; Scojen Institute of Synthetic Biology, Reichman University, Hauniversita 8, Herzliya, 4010101, Israel

## Abstract

Gene fusions are nucleotide sequences formed due to errors in replication and transcription control. These errors, resulting from chromosomal translocation, transcriptional errors or trans-splicing, vary from cell to cell. The identification of fusions has become critical as key biomarkers for disease diagnosis and therapy in various cancers, significantly influencing modern medicine. Chimeric Transcripts and RNA-Sequencing database version 8.0 (ChiTaRS 8.0; http://biosrv.org/chitars) is a specialized repository for human chimeric transcripts, containing 47 445 curated RNA transcripts and over 100 000 chimeric sequences in humans. This updated database provides unique information on 1055 chimeric breakpoints derived from public datasets using chromosome conformation capture techniques (the Hi-C datasets). It also includes an expanded list of gene fusions that are potential drug targets, and chimeric breakpoints across 934 cell lines, positioning ChiTaRS 8.0 as a valuable resource for testing personalized cancer therapies. By utilizing text mining on a curated selection of disease-specific RNA-sequencing data from public datasets, as well as patient blood and plasma samples, we have identified novel chimeras—particularly in diseases such as oral squamous cell carcinoma and glioblastoma—now catalogued in ChiTaRS. Thus, ChiTaRS 8.0 serves as an enhanced fusion transcript repository that incorporates insights into the functional landscape of chimeras in cancers and other complex diseases, based on liquid biopsy results.

## Graphical Abstract

**Graphical Abstract:**
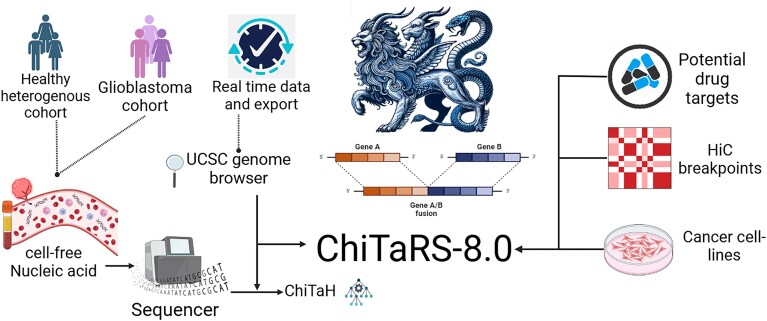

## Introduction

Eukaryote RNA transcripts are transcribed from more than one genomic locus consisting of introns and exons that are further processed into a mature messenger RNA. Fusion products are created from complex splicing events at the RNA level or as a result of chromosomal translocation (1–8). Fusion transcripts were initially identified in haematological malignancies but have since been associated with a wide range of solid tumours, including glioblastoma, melanoma, head and neck carcinomas, tumour microbiome and complex autoimmune diseases (5,9–12). Moreover, distinct genomic loci on different chromosomes produce fusion transcripts through trans-splicing, genomic rearrangement events or as read-through transcripts. Thus, the use of disease-specific large sample size can eliminate errors in their detection as artifacts (13–15).

A challenge arises when RNA-level fusions are complex to identify due to the variability in breakpoint positions and lower abundance compared with traditional DNA-level fusions. Integrating whole-genome sequencing and RNA-sequencing (RNA-seq) data has improved the accuracy of detecting fusion transcripts and distinguishing true fusion events from technical artifacts (16–19).

By employing rigorous filtering and validation processes, artifacts can be eliminated, and several studies have successfully identified in-frame fusion transcripts that produce novel fusion proteins with the potential to drive cancer as ligand-free kinases (20). Kinase fusion products of ALK, RET, ROS1 and FGFR-1/2/3, have been detected in various types of cancer, including glioblastoma, melanoma and carcinomas of the head and neck, and they are important therapeutic targets (21,22). Similarly, transcription factor fusion transcripts can promote tumourigenesis and induce changes by interacting with the genome like EWSR1–FLI1 in Ewing sarcoma (23–25). Thus, in Ewing sarcoma, therapeutic targeting of the oncogenic driver EWSR1-FLI1 may be done through inhibition of the FACT complex (26). Therefore, many fusion products are promising candidates as drug targets (27). Gene fusions containing kinases across chimeric transcripts have been included since Chimeric Transcripts and RNA-Sequencing database version 5.0 (ChiTaRS 5.0) (28). Moreover, the kinase fusions-focused database aimed at cell lines like KuNG FU, is a publicly available utility focused at creating database of fusion transcripts as therapeutic drug targets (29).

### ChiTaRS—a clinical interface for short-listing disease specific chimeric transcripts

The ChiTaRS database was developed to provide a comprehensive database of the chimeric transcripts and sequences, supporting researchers studying the genes involved in their formation, occurrence and functional significance in diseases. Starting from its earlier versions, ChiTaRS expanded to incorporate data from eight organisms, including *Homo sapiens, Mus musculus* and *Drosophila melanogaster*, with evidence from RNA-seq and other experimental techniques. The older versions of ChiTaRS, such as 2.1 and 3.1, introduced numerous improvements in both content and functionality (30–32). ChiTaRS 2.1, for instance, added over 29 000 chimeric transcripts, with 333 confirmed by RNA-seq reads mapping at the chimeric junction sites, while ChiTaRS 3.1 further expanded the collection to over 34 000 chimeric transcripts (Table 1). In addition to cataloguing transcripts, these versions also included a database of over 11 000 cancer breakpoints and the introduction of the Chimeric Protein–Protein Interaction (ChiPPI) network, which predicts the interactions of fusion proteins (28,30–32).

**Table 1.** Comparison of the past and current versions of ChiTaRS

Content	ChiTaRS	ChiTaRS 2.1	ChiTaRS 3.1	ChiTaRS 5.0	ChiTaRS 8.0
Collection of fusion genes	16 282 (total), 9379 *H. sapiens*	29 164 (total), 20 753 *H. sapiens*	34 992 (total), 24 608 *H. sapiens*	111 582 (total), 66 243 *H. sapiens*	47 445 ChiTaRS validated, 4745 FusionDB, 316 Mitelman’s database and NCBI-derived chimeras
Drug targets	No	No	No	680	5540 (982 drug targets, 4559 predicted)
Hi-C (3D chromatin contact maps) chimeras	No	No	No	5597	1055 (includes 11 cell lines and diseases, across 1055 libHiCs)
RNA-seq verified chimeras	175	337	435	937	High confidence chimeras applied across three diseases (50 RNA-seq and 16 cfDNA OSCC, 33 glioblastoma and 24 psoriasis)
Disease breakpoints	1280	1428	11 714	23 167	22 000
Manually verified breakpoints	456	1428	10 285	11 650	11 500
Sense–antisense chimeras	No	6044	6485	7521	3910
Chimeras identified across cell lines	No	No	No	2411	5956 with 1998 with frequency of 1% across 934 cell lines
Chimeras across healthy population	No	No	No	No	2066 chimeras across 199 healthy individuals
Links to external data	No	No	GeneCards (33–35), OMIM and PubMed	GeneCards, OMIM, PubMed, Ensembl, SNOMED CT and RefSeq	NCBI Nucleotide, GeneCards and Cellosaurus (36)
Discontinued					Multi-organism comparison
Future updates					Codability score, AlphaFold3 structure (37), ChiPPI with drug gene interaction from DGIdb (38) and non-human organisms with complete genomes

ChiTaRS 5.0 expanded the overall prediction of sense–antisense (SAS) fusion transcripts (Table 1). In the newest ChiTaRS 8.0 version, the focus has shifted exclusively to *H. sapiens*, discontinuing support for other organisms and centring on the fusions as a clinical tool. For the first time, we have introduced glioblastoma (brain cancer) RNA-seq data, validated through polymerase chain reaction (PCR) in 10 patients brain biopsies and liquid biopsy samples, along with oral squamous cell carcinoma (OSCC) [and cell-free DNA (cfDNA) data from blood samples of 30 patients along with 192 public RNA-seq datasets]. This enhancement makes ChiTaRS 8.0 a viable liquid biopsy tool for detecting fusion transcripts in glioblastoma and OSCC. Additionally, we have integrated healthy RNA-seq samples with a frequency filter, allowing users to exclude low-confidence chimeric transcripts based on supporting evidence. ChiTaRS 8.0 also features a ChimeraID search function integrated with the NCBI Nucleotide database. This comprehensive database serves as a valuable resource for studying the impact of chimeric RNAs in cancer, their evolutionary conservation across species and their potential to produce novel proteins with altered functional properties. Researchers can now explore protein–protein interaction networks affected by these fusions, which may provide insights into cancer progression, as well as nove fusions in complex autoimmune diseases.

### A user-friendly and versatile database user interface and navigation

ChiTaRS 8.0 introduces a highly user-friendly, guided interface that simplifies the process of data exploration. Each search function is accompanied by clear, detailed descriptions, helping users understand the purpose and functionality of every button and feature. This guided approach ensures that even new users can navigate the database easily. Each section header is designed as a clickable hyperlink, directing users to the corresponding tabulated content. This allows for seamless navigation and provides users with a complete overview of available data in just a few clicks. The interface is built to minimize the learning curve, making it accessible for researchers at various levels of expertise, from novices to experienced bioinformaticians.

In addition to the intuitive design, ChiTaRS 8.0 includes extensive references to older versions of the database (see also Supplementary Table S1). These references provide valuable historical context by detailing previous features, comparing similar databases and tracing the evolution of gene fusion nomenclature over time. This ensures that users familiar with older versions of ChiTaRS can easily adapt to the current interface, while also benefiting from insights into how the database has evolved and expanded over the time.

### Database architecture and focus

ChiTaRS 8.0 is a direct, unfiltered, HTML-based database, optimized for speed and accessibility. The open nature of the database ensures that users can access all content without any restrictions or filtering, providing complete transparency in the available data. The primary focus of ChiTaRS 8.0 is on human gene fusions, an area of critical importance for cancer research and the study of genetic diseases. The current version of ChiTaRS 8.0 contains more than 100 000 human chimeras, including an extensive catalogue of 47 445 manually curated chimeric transcripts, a third of which have been identified from RNA-seq data. These transcripts represent various types of gene fusions that can have significant implications in oncogenesis and other biological processes.

To enhance the breadth and depth of the database, ChiTaRS 8.0 has integrated 4745 additional chimeras from FusionDB, another widely used fusion transcript database (39). In addition, 186 fusions and fusion variants sourced from the NCBI database, specifically through Mitelman’s database, have been added (https://mitelmandatabase.isb-cgc.org/mb_search) (40). This combination of curated content ensures that ChiTaRS 8.0 remains one of the most comprehensive and up-to-date resources for human gene fusions, covering a wide array of fusion events from multiple sources.

### Data curation and validation tools

The data in ChiTaRS 8.0 are meticulously curated to ensure accuracy and reliability. Each entry is cross-referenced with existing literature, allowing users to trace the origin of the fusion transcripts back to peer-reviewed research. This literature-based curation guarantees that the database includes only well-documented gene fusions, reducing the risk of incorporating false positives or unverified entries (Supplementary Table S2). Additionally, the data from RNA-seq and other databases are regularly updated to reflect the latest findings in the field, ensuring that ChiTaRS 8.0 remains at the forefront of gene fusion research.

To assist users in validating and analysing gene fusions, ChiTaRS 8.0 incorporates powerful bioinformatics tools such as BLAT (BLAST-Like Alignment Tool) and BLAST (Basic Local Alignment Search Tool) (41,42). These tools allow users to perform sequence alignment and comparison, enabling the identification of homologous sequences and the confirmation of gene fusion events. By integrating these tools directly into the database, ChiTaRS 8.0 simplifies the workflow for researchers, providing a one-stop solution for fusion transcript discovery, validation and analysis.

### Applications and benefits for researchers

ChiTaRS 8.0 is a versatile tool designed to meet the needs of researchers working on human gene fusions in various biological and clinical contexts. It is particularly valuable for cancer research, where gene fusions often play a key role in tumour development and progression. The extensive catalogue of human chimeric transcripts, combined with curated data from multiple sources, positions ChiTaRS 8.0 as critical resource for understanding the molecular underpinnings of cancer and other genetic diseases.

By offering direct access to unfiltered, high-quality data, ChiTaRS 8.0 allows researchers to explore fusion transcripts without the need for complex preprocessing or filtering. The integration of user-friendly search functions, detailed descriptions and robust bioinformatics tools ensures that researchers can efficiently analyse and interpret data. The database’s comprehensive scope, combined with its ease of use, makes it an ideal platform for both large-scale genomic studies and targeted investigations into specific fusion events.

ChiTaRS 8.0 also has potential applications in clinical diagnostics, particularly in the context of liquid biopsy (43–46). The database’s focus on human gene fusions, validated through RNA-seq data, makes it well-suited for identifying fusion events that could serve as biomarkers for cancer or other diseases. Researchers and clinicians alike can use the data to identify novel fusions with clinical relevance, potentially aiding in the development of diagnostic tools or targeted therapies.

In addition, ChiTaRS 8.0 now incorporates healthy RNA-seq samples with a frequency filter that enables users to eliminate low-confidence chimeric transcripts based on supporting evidence. This feature ensures that only high-confidence fusions are presented, improving the reliability of downstream analyses.

### New features in ChiTaRS 8.0

Several new features have been introduced in ChiTaRS 8.0 to enhance its functionality and user experience. One notable addition is the ChimeraID search functionality, which allows users to query the NCBI Nucleotide database directly through ChiTaRS. This feature streamlines the process of identifying and confirming chimeric transcripts, further expanding the capabilities of the platform. The integration of this feature ensures that users can easily access external databases without leaving the ChiTaRS 8.0 interface, saving time and simplifying the research process.

**Figure 1.**
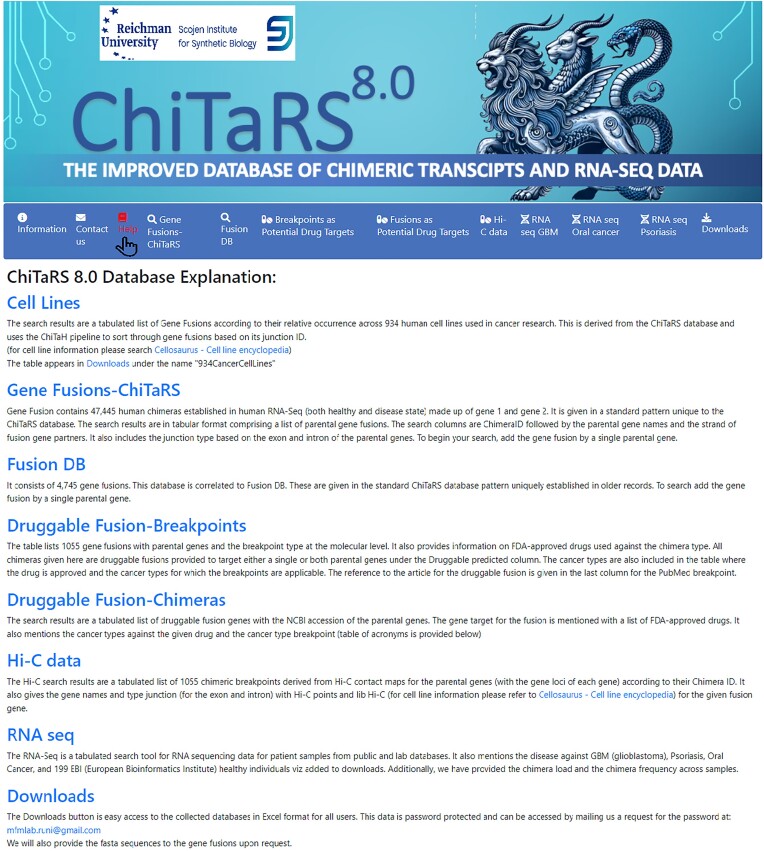
Help Tab to guide navigation with information and hyperlinks.

Overall, ChiTaRS 8.0 represents a significant advancement over previous versions, offering a more comprehensive, user-friendly and powerful tool for studying human gene fusions. Its combination of curated data, integrated analysis tools and new features make it a valuable resource for researchers across multiple disciplines. The ‘Help’ section (Figure 1) provides a comprehensive overview of previous publications related to earlier versions of ChiTaRS, serving as a reference and offering additional guidance for navigating the site and its data. We have also included an acronym table for the cancer genome atlas (TCGA) types, which can be accessed in the ‘Potential Drug Targets’ tabs for Breakpoints and Chimera searches. For detailed information about cell line types, a link is available under ‘Help > Cell Lines’, enabling users to select specific cell lines for chimera detection. The full list of cell lines can be downloaded via the ‘Downloads’ hyperlink.

### Integrated with large disease cohorts

ChiTaRS 8.0 is now integrated with extensive disease cohorts carefully curated from publicly available and proprietary RNA-seq datasets. These cohorts include a wide array of patient samples, representing different disease types and stages, providing a robust dataset for the analysis of gene fusions. The RNA-seq data are drawn from both online repositories, such as PanCancer, TCGA and offline sources, ensuring comprehensive coverage of relevant clinical samples (47–49). Each dataset has undergone rigorous curation to ensure high quality, including checks for data integrity and consistency across platforms. By incorporating both public and in-house data, ChiTaRS 8.0 provides a unique resource for studying gene fusions across diverse patient populations and disease contexts.

### Healthy cohorts and heterogeneous populations

In addition to disease-specific data, ChiTaRS 8.0 includes lists of chimeric transcripts identified in healthy cohorts. This inclusion of healthy RNA-seq samples allows users to compare fusion transcripts found in disease settings against those present in non-diseased individuals, helping to distinguish pathological fusions from those that may occur naturally as lowly expressed trandcripts. The heterogeneous population data, comprising individuals from different ethnic backgrounds, age groups and health status, adds another layer of complexity to the dataset (50–52). This diversity enables researchers to explore how gene fusions vary across different demographic factors, providing valuable insights into the biological significance and potential clinical relevance of specific fusion events.

### New data and extensions

ChiTaRS 8.0 has been significantly expanded with new data and features. One key addition is the integration of RNA-seq data with expression frequency analysis. The frequency of each chimeric transcript is calculated across patient samples, providing insights into the prevalence or rarity of fusion events within specific cohorts. This analysis is available for both in-house cfDNA sequencing data, and large public RNA-seq datasets, enabling users to compare fusion transcript prevalence in cfDNA with that in tissue-derived RNA expression levels. The frequency data also help to prioritize high-confidence fusion events for further investigation or potential clinical applications.

The database of fusion transcripts specific to cancer cell lines has been expanded. The ‘Cancer Cell Line’ can be downloaded from the ‘Help’ hyperlink or ‘Download’ button. It represents an expanded collection of high-confidence reference-based chimeras detected using ChiTaH and the 43 466 chimera dataset (https://github.com/Rajesh-Detroja/ChiTaH), our in-house, fast and accurate fusion transcript reference-based detection algorithm (53). Currently, the database for ChiTaH and ChiTaRS are merged into a larger database and several chimeric transcripts has been added to its repertoire for future reference.

For example, ChiTaRS 8.0 includes RNA-seq data from 192 well-selected whole transcriptome datasets specifically focused on OSCC and related cancer types. These include head and neck squamous cell carcinoma (HNSC), oesophageal squamous cell carcinoma and tongue cancer subtypes of OSCC. In addition to primary tumour data, ChiTaRS 8.0 also incorporates RNA-seq data from OSCC-derived cervical lymph node cell lines, such as HSC-3, HSC-4, KYSE-150, and SAS fusions. These datasets offer a comprehensive view of gene fusions associated with OSCC, providing researchers with the ability to compare fusion events across different cancer subtypes and cell lines.

### Extensions for the liquid biopsy applications

Liquid biopsy has emerged as a revolutionary approach in cancer diagnostics, complementing traditional methods with a less invasive alternative. This technique leverages various bodily fluids, including blood, urine, saliva, cerebrospinal fluid and pleural effusions, to assess a range of cancer biomarkers (54). This technique analyses various cancer-related substances found in body fluids such as circulating tumour cells, cfDNA, circulating tumour DNA (ctDNA), circulating microRNAs and extracellular vesicles. Each of these substances can provide important information about the presence and behaviour of cancer in the body (55). The specific biomarkers detected in liquid biopsy can include a wide range of genetic and molecular changes such as DNA mutations, gene deletions or amplifications, gene fusions, DNA methylation patterns, cancer-specific microRNAs, proteins and metabolites (56). Recent innovations in liquid biopsy technologies, particularly digital PCR and Next Generation Sequencing, have markedly improved the precision and sensitivity of ctDNA analysis. These advanced techniques facilitate earlier cancer detection, enable high-resolution monitoring of tumour DNA dynamics, allow for the identification of minimal residual disease and provide real-time insights into cancer evolution (57).

In ChiTaRS 8.0, large RNA-seq datasets are directly compared with in-house cfDNA samples from 30 OSCC patients, allowing for cross-comparison between fusion events identified in plasma (cfDNA) and those found in solid tumour tissues or cell lines. This integration of liquid biopsy data with RNA-seq datasets opens up new possibilities for studying the dynamics of gene fusions in a minimally invasive manner, potentially aiding in the development of liquid biopsy tools for cancer diagnosis and monitoring (43–47). The inclusion of cfDNA data is especially valuable for capturing fusion events that may not be detectable in bulk tumour sequencing, offering insights into tumour heterogeneity and clonal evolution (43,44,58,59).

By comparing the fusion transcript frequencies in cfDNA samples with those observed in large RNA-seq cohorts, researchers can identify fusions that are consistently detected across different platforms and sample types, particularly for mitochondrial DNA fusions. This aids in validating fusion candidates as potential biomarkers and ensures that only highly reliable, biologically significant fusions are prioritized for additional research.

### OSCC cancers

We conducted an in-depth analysis of oral cavity cancers, focusing specifically on OSCC. As part of our study, we received 30 biopsy samples from Rabin Medical Center (Petah-Tikva, Israel), where we identified several unique chimeric transcripts specific to OSCC. In addition to these samples, 192 OSCC and 199 heterogeneous healthy control RNA-seq datasets from publicly available sources were analysed with ChiTaH (53).

**Figure 2.**
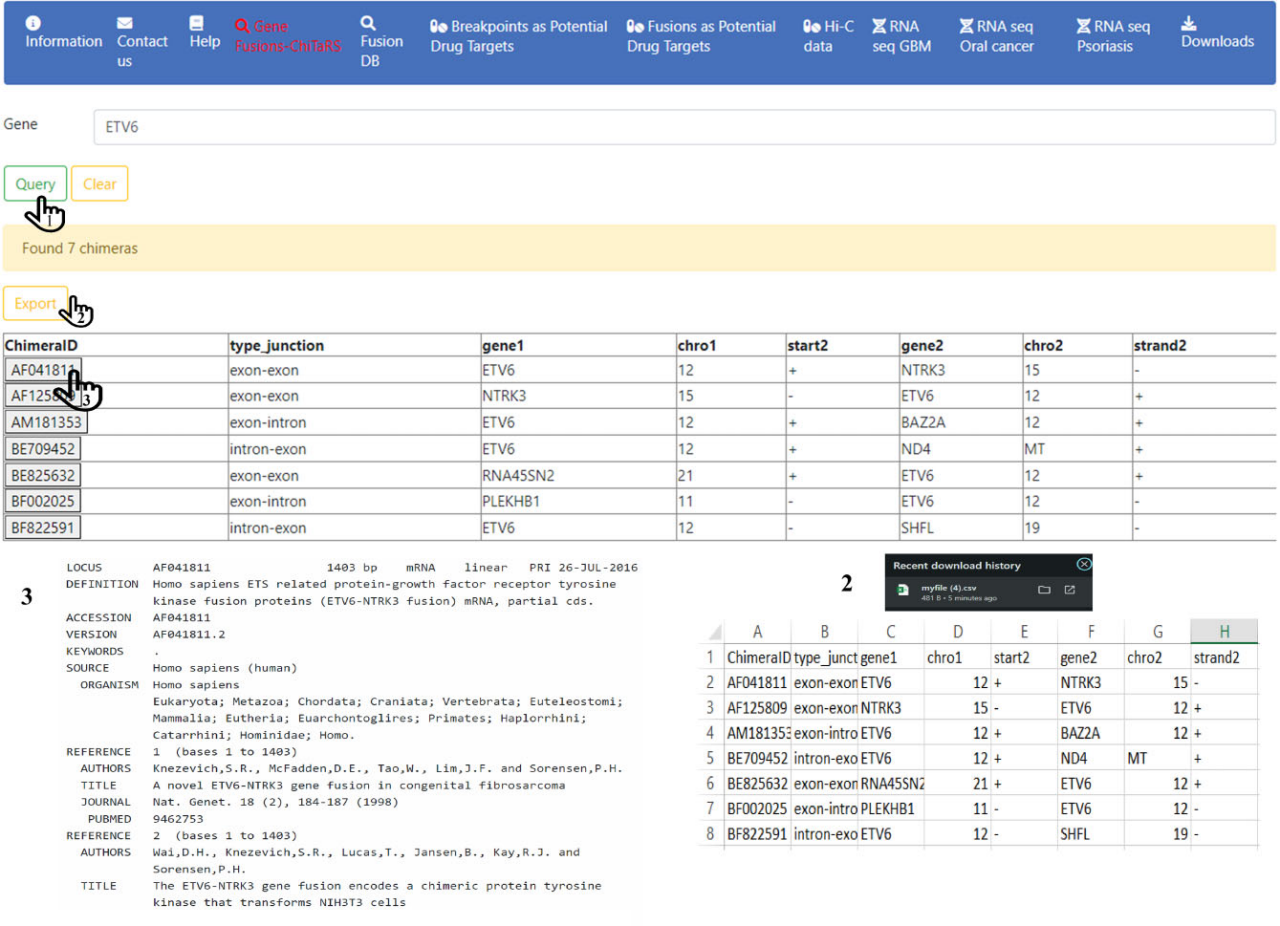
ChimeraID is a key to find and test the NCBI record in GenBank. GeneCards entry link has been added as a hyperlink for the parental genes in every fusion.

Our comparative analysis revealed a significant enrichment of unique chimeras in the OSCC samples compared with the normal controls (Table 2). These chimeric transcripts have potential clinical utility as biomarkers for the early diagnosis of primary OSCC, and for detecting disease recurrence. PMS2P9-CCDC146 chimera has been identified as a high-confidence chimera in our public dataset study and has been reported as a novel putative biomarker for HNSC carcinomas by Valerio *et al.* (60). Similarly, a chimeric transcript with the parental genes COL7A1-UCN2 (Table 2) ) was reported among laryngeal cancer samples by Tao *et al.* (61). Consequently, utilizing our healthy fusion transcripts and a large sample size of OSCC RNA-seq enabled us to distinguish between normal and cancerous tissues. This highlights their promise in improving diagnostic accuracy and potentially guiding personalized treatment strategies. The identified chimeras have been uploaded to the latest ChiTaRS 8.0 database update, contributing valuable data for further research and offering insights into their use in early diagnostics and monitoring of OSCC (Figure 2).

**Table 2.** RNA-seq data for 192 OSCC samples from GEO datasets with 10 highly abundant chimeras observed in RNA-seq

ChimeraID	Frequency in healthy	Chimera type	Gene1	Gene2	JunctionID	Frequency	Average junction count
CV377015	1.01	exon-exon	ATP6	ATP8	CV377015:132–163	80.73	9.25
FY211193	1.51	exon-exon	UCN2	COL7A1	FY211193:81–112	80.21	15.54
HY370423	9.55	exon-exon	LRRC75A	SNHG29	HY370423:408–439	92.19	16.98
CV354006	3.52	exon-intron	ATP6	ATP8	CV354006:201–232	93.23	88.96
BY800312	5.53	intron-exon	AP001453.2	PPP1R14B-AS1	BY800312:294–325	85.42	7.51
DA196762	0.5	intron-exon	FLJ31356	FOSL2	DA196762:191–222	81.25	6.33
DA620360	Not found	intron-exon	GSN	RAB14	DA620360:271–302	80.21	3.33
DB489365	Not found	intron-exon	GPR75-ASB3	ASB3	DB489365:169–200	82.29	2.76
HY089930	7.54	intron-exon	PDCD4	PDCD4-AS1	HY089930:271–302	80.73	4.97
N28607	1.51	intron-exon	BOLA2B	SMG1P2	N28607:148–179	80.21	4.89

Column 1 is the ChimeraID is part of our tabulated data on Gene Fusions – ChiTaRS, which can be used as a reference to acquire additional information like nucleotide sequence, study, sample and study group from NCBI; column 2 is the frequency of the chimera in healthy samples at <10% and not found; columns 3–6 are derived for chimera information and junctionID for RNA-seq and are part of the ChiTaRS table; column 7 is the occurrence across 192 samples; and column 8 is the average read count of the chimera across the sample. CV354006 highlighted for Figure 2.

### Chimeras in glioblastoma samples

Glioblastoma is an aggressive and malignant form of brain cancer, characterized by rapid growth and resistance to conventional treatments. As part of our study, we received human brain biopsy samples from Beilinson Hospital (Rabin Medical Center), enabling us to conduct deep RNA-seq using 10 patients samples, with a minimum coverage of 20 million reads each, followed by the cfDNA analyses from the blood samples (see Supplementary Material).

**Figure 3.**
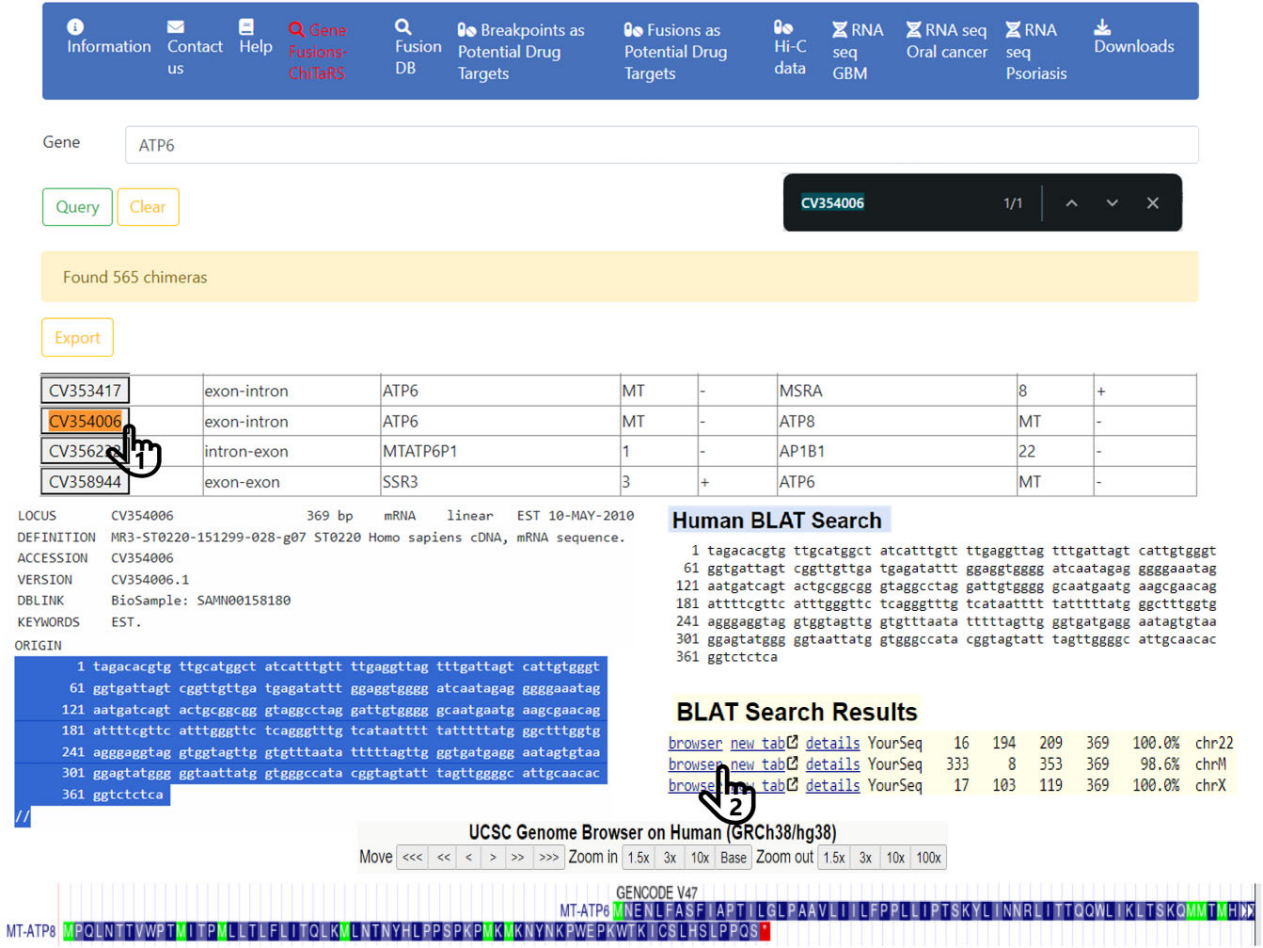
The highlighted is a high confidence chimera from RNA-seq of OSCC samples (see Table 3). Additional parameters for ChiTaRS 8.0 utilization (top panel), NCBI search by click; a FASTA link (left panel); BLAT human genome search and browser (right panel) and the UCSC genome view (bottom panel).

Through this analysis, we were able to identify several highly abundant chimeric transcripts, as shown in Figure 3 and Table 3. The parental genes, such as GFAP, TTTY18 and ATP5F1B (Figure 3), have been studied as individual biomarkers due to their expression and roles in glioblastoma (62–65). Glioblastoma disease progression is influenced by oxidative stress associated with these parental genes (65,66). To ensure accuracy PCR is used to confirm these results with positive controls. Importantly, these chimeras hold potential to be employed in liquid biopsy applications, offering non-invasive biomarkers for glioblastoma detection and monitoring (Table 3). The validated chimeras have been incorporated into the latest ChiTaRS 8.0 update, enriching the database with novel data specific to glioblastoma and providing a valuable resource for ongoing research and potential clinical applications.

**Table 3.** Sequencing results for highly abundant chimeras in glioblastoma cfDNA samples

Chimera ID	Chimera type	Gene 1	Gene 2	Frequency in healthy	Frequency in glioma
BE071135	exon-exon	ND4	ND4L	1.01%	100%
BF819282	exon-exon	ATP5F1B	CLU	4.52%	100%
CV370968	exon-exon	ND4	ND4L	NOT FOUND	100%
CV354006	exon-intron	ATP6	ATP8	3.52%	100%
BM820741	exon-intron	ATP6	C0 × 3	18.09	100%
AL583020	exon-exon	COX3	ATP6	18.09/1.59 reads	90%/15.4 reads
CV377015	exon-exon	ATP6	ATP8	1.01%	100%
CV413189	exon-exon	ND6	ND5	NOT FOUND	90%
EC443603	exon-exon	ND5	ND6	NOT FOUND	90%
BF343429	exon-intron	GFAP	TTTY18	NOT FOUND	90%

### Future plans

As part of our ongoing efforts to enhance ChiTaRS 8.0, we are actively exploring the integration of a ‘digital hospital’ model. This model aims to streamline the addition and updating of data in a highly organized, tabulated format. This initiative is part of a broader harmonization strategy (39,40) to standardize data across multiple platforms and ensure seamless updates. The digital hospital model will allow for the real-time incorporation of clinical data and make it easier to add new datasets and fusion transcript information as they become available. By utilizing this format, researchers will be able to access up-to-date, curated gene fusion information that can be dynamically updated based on new clinical findings.

One of our key future objectives is the integration of ChiPPI and fusions as potential drug targets into ChiTaRS 8.0. This expansion will enable the database to serve as a predictive tool for guiding targeted drug testing, particularly for cancer therapies. By leveraging the gene fusion data already stored in ChiTaRS 8.0, along with drug interaction predictions from ChiPPI, researchers will gain access to a comprehensive resource to identify drug targets and test therapeutic strategies. We are working to enhance this resource with our expanding dataset of gene fusions identified across 934 cancer cell lines, which will be available for download under the ‘Download’ button. This extensive collection of fusion data will help researchers investigate how these chimeric transcripts influence cancer progression and drug response across different cell lines, potentially aiding in the development of personalized medicine approaches.

One of our goals was leveraging the Drug Gene Interaction Database (DGIdb), to comprehensively evaluate whether the coding potential of fusion transcripts introduces potential targets (38,67). This involves systematically assessing whether the fusion proteins retain domains—such as kinases or receptors—that are currently targeted by small-molecule inhibitors or monoclonal antibodies. Some gene fusions may increase drug sensitivity, particularly to targeted therapies like kinase inhibitors. Future research should aim to identify and characterize which fusion events enhance sensitivity to specific treatments, enabling the use of precision medicine approaches. By integrating genomic and functional data, we can tailor treatment strategies to exploit the vulnerabilities created by these fusion events. Such insights will be particularly valuable in guiding clinical decisions and improving patient outcomes by matching therapies to the genetic makeup of individual tumours.

We also plan to expand ChiTaRS 8.0 by adding new data every 6 months. Each new dataset will undergo rigorous validation using BLAT and BLAST search tools to ensure accuracy and relevance (41,42). By maintaining this regular update schedule, we aim to keep the database at the cutting edge of gene fusion research, ensuring it remains a valuable tool for the scientific community. Furthermore, we intend to broaden the scope of the database by incorporating fusion transcripts from an even broader array of cancers and tissue types, providing researchers with a more comprehensive overview of gene fusions in both rare and common malignancies.

An important aspect of our future work will be the assessment of the codability of chimeric transcripts. Determining the coding potential of these transcripts is essential for understanding their biological significance, as not all chimeras result in functional proteins (61,68,69). By integrating tools and algorithms that predict codability, we will allow researchers to prioritize fusion transcripts that are likely to produce functional proteins, which may be critical for disease progression or therapeutic targeting.

## Discussion

The expansion of ChiTaRS 8.0 into a more dynamic and clinically relevant tool reflects our commitment to bridging the gap between basic research and clinical application. The integration of a digital hospital model, combined with data on drug targets from ChiPPI, will make ChiTaRS 8.0 a powerful platform for both academic researchers and clinicians. By providing access to curated gene fusion data across hundreds of cancer cell lines, we can help drive new discoveries in cancer biology and support the development of targeted therapies. Our approach aligns with the growing trend towards personalized medicine, where understanding the unique genetic landscape of each patient’s tumour can inform more precise and effective treatment strategies.

The incorporation of drug target predictions is especially promising, as it could directly inform clinical decision-making. As more therapies targeting specific gene fusions enter clinical trials, the ability to identify abundant fusions as drug targets in patient samples could lead to more personalized treatment regimens, improving outcomes for cancer patients. ChiTaRS 8.0, combined with predictive tools like ChiPPI, could serve as a key resource for identifying candidate fusions for drug testing, guiding research into new therapeutic avenues.

Moreover, the regular inclusion of new data and the validation of these additions through BLAT and BLAST searches ensures that the database remains up-to-date and scientifically reliable. Our focus on validating fusion transcripts before including them in the database is crucial for maintaining the high quality of the data, reducing the risk of false positives and ensuring that researchers can trust the information they retrieve from ChiTaRS 8.0. The incorporation of codability assessments is another step towards ensuring that the most biologically relevant chimeric transcripts are prioritized for research and clinical application.

In the long term, ChiTaRS 8.0 aims to serve as a hub for both research and clinical applications, supporting investigations into gene fusions across various diseases and helping to identify novel biomarkers for diagnostic or therapeutic use. As the field of gene fusion research continues to grow, we remain committed to expanding and refining ChiTaRS 8.0 to meet the evolving needs of the scientific and medical communities.

## Supplementary Material

gkae1126_Supplemental_File

## Data Availability

All the data are available at http://biosrv.org/chmb/information.

## Notes

Present address: Milana Frenkel-Morgenstern, Scojen Institute of Synthetic Biology, Reichman University, Hauniversita 8, 4010101, Herzliya, Israel.
