# Supplementary material for: ChiTaRS 8.0: the comprehensive database of chimeric transcripts and RNA-seq data with applications in liquid biopsy
Source: Nucleic Acids Res. 2024 Dec 16;53(D1):D1302–12. doi: 10.1093/nar/gkae1126 (PMC11701575; doi:10.1093/nar/gkae1126)
Supplement: gkae1126_Supplemental_File [file gkae1126_supplemental_file.pdf]

Supplementary Material

***Title:** ChiTaRS-8.0: The comprehensive database of Chimeric Transcripts and RNAseq data with applications in Liquid Biopsy.*

Authors: Dylan DSouza[1#], Lihi Bik[2#], Olawumi Giwa[1#], Shahaf Cohen[2], Hilit Levy Barazany[2], Tali Siegal[3], Milana Frenkel-Morgenstern[1,2,4*]

Affiliation:

[1] Azrieli Faculty of Medicine, Bar Ilan University, Henrieta Szold 8, Safed Israel.

[2] Scojen Institute of Synthetic Biology, Reichman University, Hauniversita 8, Herzliya, Israel.

[3] Rabin Medical Center (Beilinson Campus), Zeev Jabotinsky St 39, Petah Tikva, Israel.

[4] **Current affiliation:** Scojen Institute of Synthetic Biology, Reichman University, Hauniversita 8, Herzliya, Israel.

[#] Equally contributed to this work.

***Corresponding Author:** Milana Frenkel-Morgenstern milana.morgenstern@runi.ac.il

Website: http://biosrv.org/chitars

PCR VALIDATIONS OF FUSION GENES IN TOTAL RNA AND CFDNA SAMPLES OF GLIOBLASTOMA PATIENTS' SAMPLES.

We identified the top 10 chimeric transcripts that were highly abundant and prevalent in 10 and 9 out of 10 glioblastoma patients' samples (90% frequently found in patients) submitted for whole exome sequencing while exhibiting a frequency of 5% or lower among 199 healthy samples (5% FDR). We designed appropriate primers to perform PCR to validate the presence of these fusions in glioblastoma patients' samples.

Top 10 abundant chimeric transcripts and their ID.

Chimera ID	Chimera type	Gene 1	Gene 2	Frequency @ Healthy	Frequency @ Glioma
BE071135	exon-exon	ND4	ND4L	1.01%	100%
BF819282	exon-exon	ATP5F1B	CLU	4.52%	100%
CV370968	exon-exon	ND4	ND4L	NOT FOUND	100%
CV354006	exon-intron	ATP6	ATP8	3.52%	100%
BM820741	exon-intron	ATP6	COX3	18.09	100%
AL583020	exon-exon	COX3	ATP6	18.09/1.59 reads	90%/15.4 reads
CV377015	exon-exon	ATP6	ATP8	1.01%	100%
CV413189	exon-exon	ND6	ND5	NOT FOUND	90%
EC443603	exon-exon	ND5	ND6	NOT FOUND	90%
BF343429	exon-intron	GFAP	TTTY1 8	NOT FOUND	90%

Note: We selected the chimeric transcripts with an 18.09% frequency in healthy patients, since they exhibited 1.59 (low fold change) in healthy individuals and significantly higher read counts of 15.4 (high fold change) in glioblastoma patient samples.

RESULTS

We obtained the bands associated with the 10 highly abundant chimeras as expected in 9 out 10 chimeras in 10 patients' samples.

RNA BE071135 -ND4-ND4L:
(EXPECTED PRODUCT SIZE, 192bp)

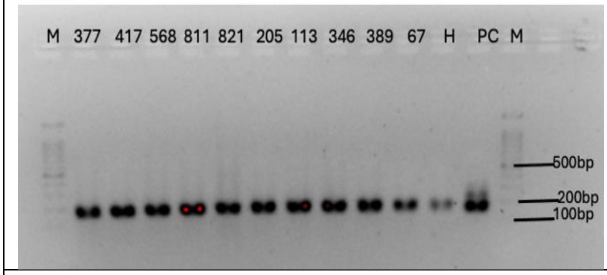

RNA BF819282 -ATPF5F1B-CLU:
(EXPECTED PRODUCT SIZE, 186bp)

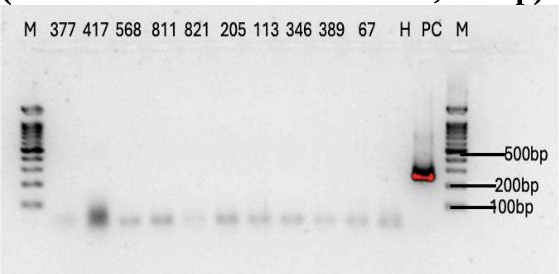

RNA CV370968 -ND4-ND4L:
(EXPECTED PRODUCT SIZE, 174bp)

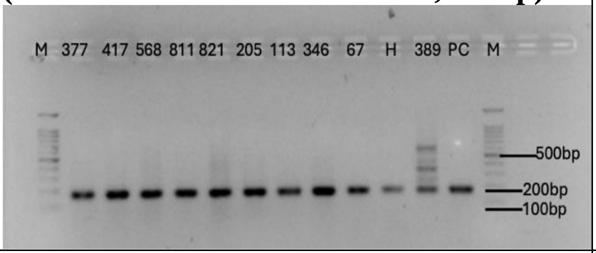

RNA CV354006 - ATP6-ATP8:
(EXPECTED PRODUCT SIZE, 337bp)

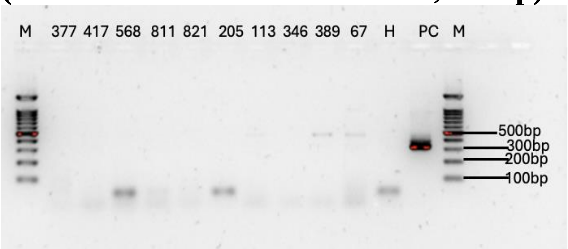

RNA BM820741-ATP6-ATP8:
(EXPECTED PRODUCT SIZE, 211bp)

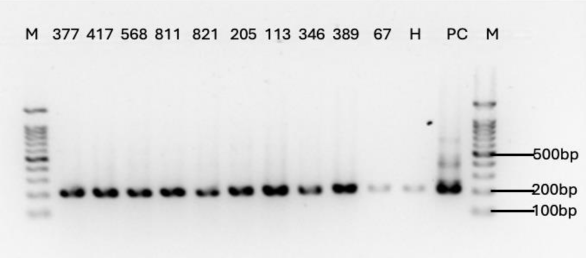

RNA AL583020 - COX3-ATP6:
(EXPECTED PRODUCT SIZE, 378bp)

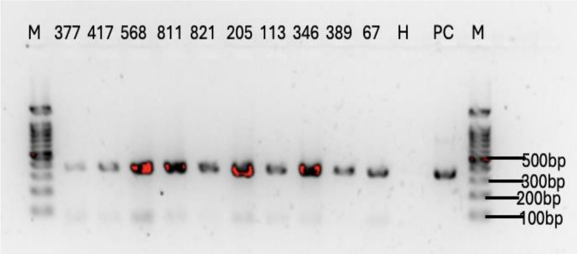

RNA CV377015 – ATP6-ATP8:
(EXPECTED PRODUCT SIZE, 191bp)

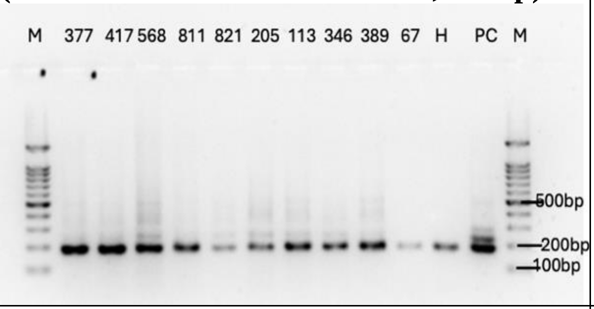

RNA CV413189 – ND6-ND5:
(EXPECTED PRODUCT SIZE, 196bp)

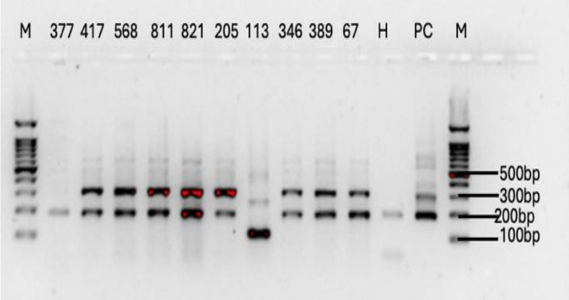

RNA EC4436303 – ND5-ND6:
(EXPECTED PRODUCT SIZE, 116bp)

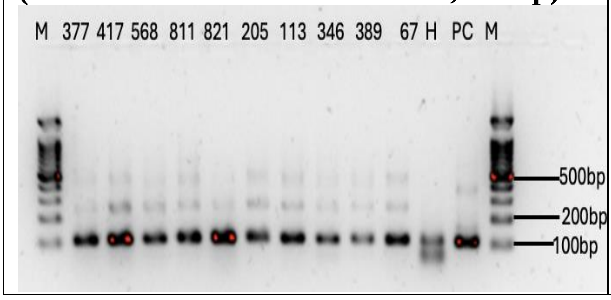

Supplementary Fig. 1: Validation of fusions in RNA samples of 10 glioblastoma patients by PCR with dedicated primers.

**CFDNA BE071135 – ND4-ND4L: (EXPECTED PRODUCT SIZE, 192bp)**	**CFDNA BF819282 – ATPF5F1B-CLU: (EXPECTED PRODUCT SIZE, 186bp)**

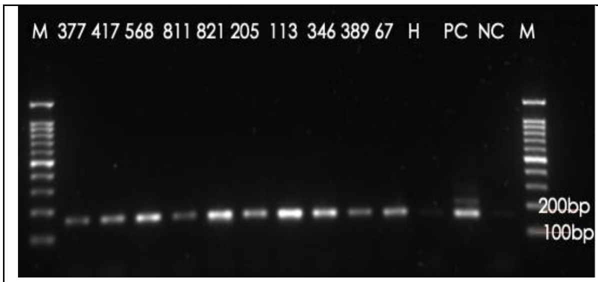

CFDNA CV370968 – ND4-ND4L: (EXPECTED PRODUCT SIZE, 174bp)

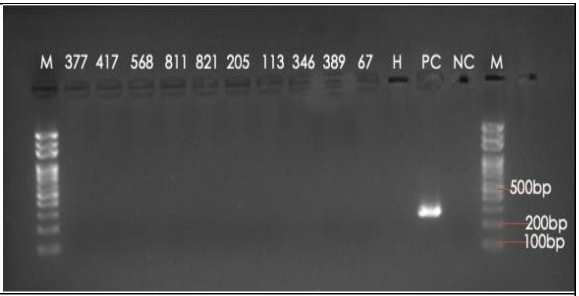

CFDNA CV354006 ATP6-ATP8: (EXPECTED PRODUCT SIZE, 337bp)

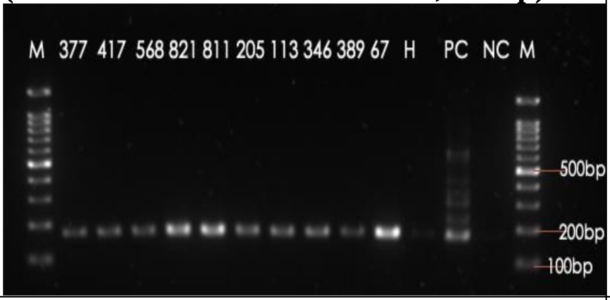

CFDNA BM820741 - ATP6-COX3: (EXPECTED PRODUCT SIZE, 211bp)

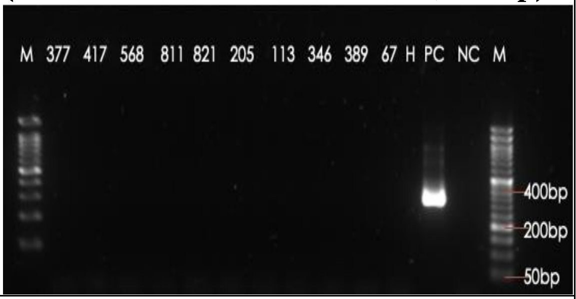

CFDNA AL583020 - C0X3-ATP6: (EXPECTED PRODUCT SIZE, 378bp)

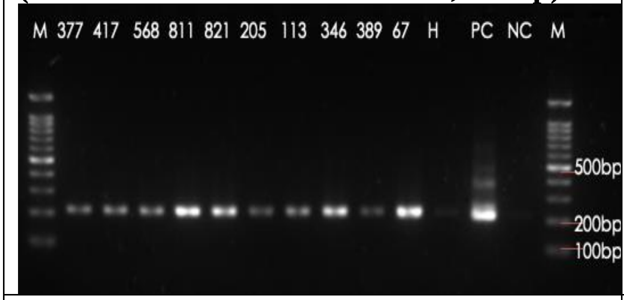

CFDNA CV377015 – ATP6-ATP8: (EXPECTED PRODUCT SIZE, 191bp)

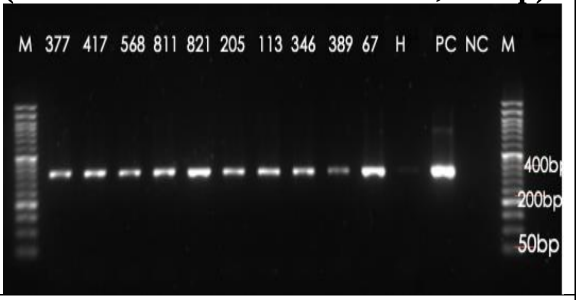

CFDNA CV413189 – ND6-ND5: (EXPECTED PRODUCT SIZE, 196bp)

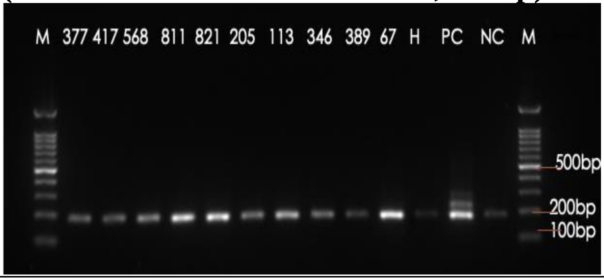

CFDNA EC4436303 – ND5-ND6: (EXPECTED PRODUCT SIZE, 116bp)

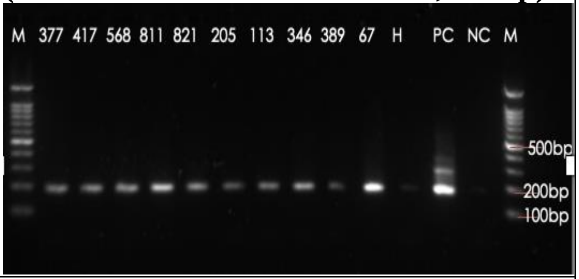

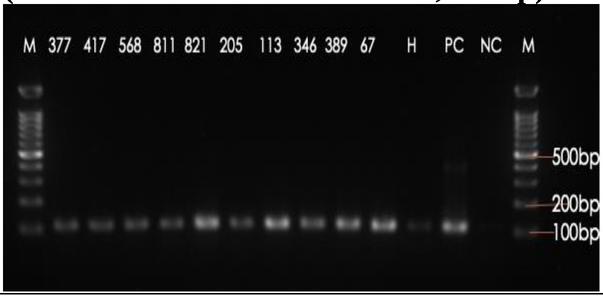

Supplementary Fig. 2: Validation of fusion in cfDNA samples in glioblastoma patients. The fusions appeared in RNA and cfDNA samples since they have incorporated mitochondrial genes that are encoded without introns. Note: Lane 377 -67: glioblastoma patients. Lane H: Healthy patients. Lane PC: Positive control. Lane NC: Negative control. Lane M: Ladder

Discussion

The results presented in this study provide valuable insights into the presence of fusion genes in glioblastoma patients. Our findings demonstrate the successful validation of several fusion genes in RNA and cfDNA samples from these patients, offering new avenues for diagnosis, prognosis, and treatment of this aggressive brain cancers.

RNA Sample Analysis

Our PCR results for RNA samples revealed successful amplification of multiple fusion genes in glioblastoma patients. Specifically, BE071135 (ND4-ND4L), BM820741 (ATP6-ATP8), AL583020 (COX3-ATP6), CV377015 (ATP6-ATP8), CV413189 (ND6-ND5), and EC4436303 (ND5-ND6) all showed bands of expected sizes in glioblastoma patient samples. Importantly, these fusion genes were either absent or present at significantly lower frequencies in healthy samples, supporting their potential as glioblastoma-specific markers.

cfDNA Sample Analysis

The cfDNA results largely corroborated our RNA findings, with BE071135, CV370968, BM820741, AL583020, CV377015, CV413189, and EC4436303 all showing amplification in cfDNA samples from glioblastoma patients. The presence of these fusion genes in cfDNA is particularly significant, as it suggests the potential for developing non-invasive liquid biopsy-based diagnostic tools for glioblastoma.

Mitochondrial Gene Involvement

A striking feature of our findings is the predominant involvement of mitochondrial genes in the validated fusion genes. Most of the identified fusions involve mitochondrial genes such as ND4, ND4L, ATP6, ATP8, COX3, ND5, and ND6. This observation suggests a potential role of mitochondrial dysfunction in glioblastoma pathogenesis, opening new avenues for research into the hypoxia and inflammation markers in glioblastoma.

Supplementary Table 1: Comparison of current databases on gene fusions and specialised databases for fusion kinases and fusion genes specific to cell lines.

Database Name	Description	Key Features	URL
**COSMIC (Catalogue Of Somatic Mutations In Cancer) [1-3]**	A comprehensive database for somatic mutations including fusion genes in cancer.	Focuses on cancer mutations and gene fusions.<br>Supports clinical research and drug development.	https://cancer.sanger.ac.uk/cosmic
**TCGA (The Cancer Genome Atlas) [4-8]**	Landmark cancer genomics program that includes fusion gene data.	Extensive cancer datasets.<br>Includes fusion genes along with expression data.	https://portal.gdc.cancer.gov/
**Mitelman Database of Chromosome Aberrations and Gene Fusions in Cancer [9, 10]**	Comprehensive resource for chromosome aberrations and fusion genes in cancer. Updated quarterly	Contains gene fusions from various cancers.<br>Focus on cytogenetic changes in cancer.	https://mitelmandatabase.isb-cgc.org/
**ChiTaRS (Chimeric Transcripts and RNA-seq Database) [11-14]**	A database of chimeric RNA transcripts in humans.	Contains fusion transcripts from cancer and normal samples.<br>Allows search by gene, potential therapy, cancer type.	http://biosrv.org/chitars
**DepMap (Cancer Dependency Map) [15, 16]**	Provides data and tools for understanding cancer vulnerabilities, including gene fusions.	Includes tools for analyzing gene dependencies in over 2,000 cancer cell lines.<br>Regular updates with new cell line data.	https://depmap.org/portal/
**KuNG FU [17]**	A kinase fusion gene database focused on cancer cell lines.	Contains manually curated kinase gene fusions from cancer cell lines.<br>Targets in-frame fusions with intact catalytic domains.	https://kungfudb.org/
**ChimerDB 4.0 [18-21]**	A database dedicated to fusion genes derived from cancer and normal samples.	Provides information on fusion gene sequences.<br>ChimerSeq includes RNA-seq and EST data from ChiTaRS$^{3.1}$<br>Annotates fusions with clinical relevance.	https://www.kobic.re.kr/chimerdb/
**FusionGDB [22, 23]**	A gene fusion annotation resource for gene fusions.	Focuses on annotation of fusion genes.<br>Provides fusion classification and biological significance.	https://compbio.uth.edu/FusionGDB2/
**dbCRID (Database of Cancer-related Fusion Genes) [24]**	An archive database with manually curated cancer-related fusion genes.	Provides experimentally validated cancer fusions.<br>Contains gene fusion frequencies.	https://web.archive.org/web/20120324085623/http://dbcrid.biolead.org/

Supplementary Table 2: The fusions analysis and validation steps.

Step	Objective	Input	Tools/Methods	Output
**1. Initial Identification**	Detect potential gene fusion events from sequencing data.	Gene fusion candidates from RNA-seq, WGS, or WES.	STAR-Fusion (RNA-seq), FusionCatcher (RNA-seq & WGS), ChiTaH (Chimeric RNA analysis).	Comprehensive list of detected fusion events.
**2. Filtering**	Remove false positives, artifacts, and common fusions.	Initial list of detected fusions.	Frequency-based Filtering, Threshold Criteria (read support, occurrence in samples, low-confidence removal).	Refined list of gene fusion candidates.
**3. Functional Annotation**	Understand the biological role of fusion partners.	Refined fusion list.	GeneCards/Ensembl (gene function, protein domains), FusionGDB (known fusion databases).	Functional annotation data for fusion pairs (gene function, domain structure, pathways).
**4. Literature and Pathway Analysis**	Investigate biological pathways and networks.	Functional annotation data.	Literature Review (PubMed, Google Scholar), MetaScape (functional enrichment), Reactome/STRING (protein-protein interactions).	Literature summaries and pathway data for the biological role of fusion genes.
**5. Coding Potential and Druggability Assessment**	Assess coding potential and therapeutic targeting.	Annotated fusion sequences.	CPAT (coding potential), PROVEAN (functional impact analysis), DGIdb (drug-gene interactions, actionable fusions).	Assessment of coding potential, domain disruption, and druggability.
**6. Experimental Validation**	Experimentally confirm fusion candidates' presence and significance.	High-confidence fusion candidates.	RT-PCR (fusion transcript validation), qPCR (expression level verification), FISH (chromosomal visualization).	Experimentally validated gene fusions with confirmed biological and clinical relevance.
